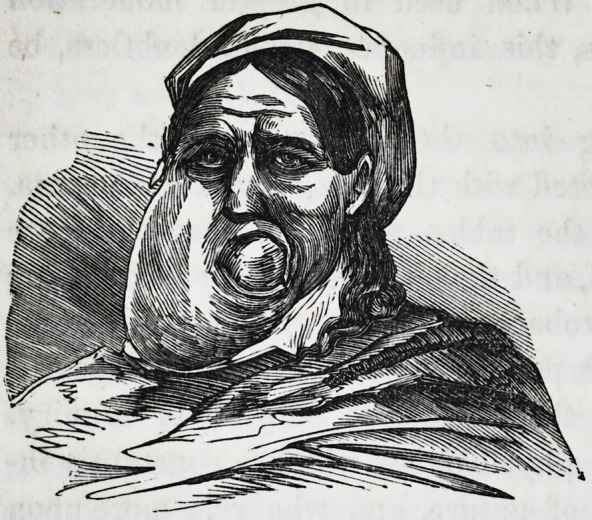# Removal of the Entire Lower Jaw for Osteo-Sarcoma

**Published:** 1857-04

**Authors:** George C. Blackman

**Affiliations:** Professor of Surgery in the Medical College of Ohio; Surgeon to the Commercial Hospital, Cincinnati, &c.


### ARTICLE X.

*Removal of the Entire Lower Jaw for Osteo-sarcoma.* By GEORGE C. BLACKMAN, M. D., Professor of Surgery in the Medical College of Ohio; Surgeon to the Commercial Hospital, Cincinnati, &c. (With a wood-cut.)

MRS. V., æt. 60, corpulent, and of excellent general health, consulted me in May last, in reference to an affection of the lower jaw, with which she had been troubled for about forty years. Its origin was attributed to an injury inflicted during the extraction of a decayed tooth on the right side. The swelling extended, at the time of my first visit, from near the external angle of the eye to the opposite ramus of the lower jaw. The mouth was enormously elongated and distorted, as represented in the wood-cut.

The tumor extended downwards below the clavicle, and at its lowest point there was an opening some two inches in diameter, through which a bloody matter was discharged in large quantities. For many years the disease was very slow in its progress, but during the past two years the latter had been much more rapid. Of late, she had experienced much difficulty in taking nourishment, and, from the pressure of the enormous mass on the side of her throat, she was often threatened with suffocation. With the exception of the fungous opening already mentioned, the integuments covering the tumor presented a natural appearance. The glands of the neck were free from disease.

I advised an operation, and urged its performance before the commencement of the hot season. She failed, however, to arrange her affairs to come to the city until the latter part of June. At her request, chloroform was administered, or rather a mixture of one part of chloroform and two of sulphuric ether. She was very readily brought under its influence, when I made an incision which commenced just in front of the ear, on a level with the right eye, and extended to the angle on the left side. It passed about an inch and a half below the border of the lower lip, and the commissure was not divided on either side. Another incision was made which included the fungous opening, and the superabundant integument which required removal. The flaps were rapidly dissected, and the bony tumor exposed. The facial arteries bled freely, but were at once controlled by pressure until the ligatures were ready to be applied. With one of Luer's small saws I divided the bone at the left angle, detached the tongue, and disarticulated the right ramus. This was rendered less difficult, as the pressure produced on the condyles by the extension of the tumor beneath the zygoma, caused an absorption of their substance so that they readily separated, and the portions remaining at the articulation were easily extracted with the forceps. As the disease had already invaded the left ramus, this was also removed from thc articulation. Retraction of the tongue, which had nearly proved fatal in several of my previous operations on the lower jaw, was guarded against at the very outset of the operation, by passing a strong cord through the organ, which was held by a trusty assistant. In this case, however, there seemed to be no tendency to any such retraction. The removal of the entire bone was accomplished in about fifteen minutes, and the patient lost no more than from six to eight ounces of blood. For an hour after the operation, (at 3 P. M.,) she seemed greatly prostrated, and until 8 o'clock that evening her pulse continued feeble. Beef-tea, wine, or brandy were regularly administered, at first through a tube, afterwards through the spout of a cup contrived for the purpose. There was no difficulty in swallowing, and her respiration was easy. She passed the night as comfortably as could have been expected, and at 8 o'clock next morning took her nourishment well, and even articulated some words distinctly. At 9 o'clock the heat was intense; no breeze was stirring, and as the sun approached the meridian, the thermometer rose in the patient's chamber to 98°. At 11 o'clock it became evident that her strength was failing, and at 1 o'clock, P. M., when the heat had attained its greatest intensity (100° Fahr.) she died without a struggle.

I was assisted in the operation by several of the first physicians of Cincinnati, among whom I may name Drs. Carrol, Fries, Dandridge, Dodge, Foster, Armor, Muscroft, &c., and I believe all agree in attributing her death to the exhaustion occasioned by the intense heat. Perhaps the anæsthetic likewise exercised a deleterious influence, but how much is due to that, it would be difficult to determine.

The tumor weighed 3½ lbs., and presented all the anatomical characteristics of osteo-sarcoma.

The removal of the entire lower jaw for necrosis has been performed by Perry, of England; Ganwesky, of Westphalia; Maisonneuve, of Paris; Pitha, of Prague, and Heyfelder, of Erlangen; also by McClellan, Carnochan, Marsh, and James R. Wood, of our own country. These cases are of interest, inasmuch as their results furnish us with illustrations of the wonderful reparative powers of nature, but they can hardly be classed with the operations for osteo-sarcoma executed by Professor Syme, Mr. Cusack, of Dublin; Mr. O'Shaughnessy, of India; by Dieffenbach, of Berlin; by Dr. Mott in the case of the negro "Prince;" by Dr. Ackley, of Cleveland, and I think I may add, by myself. In Professor Syme's case of removal of the entire lower jaw, the patient died suddenly the day after the operation, as was supposed, from suffocation produced by the retraction of the tongue. (*Contributions to the Pathology and Practice of Surgery*, p. 21.) Mr. Cusack informed me in June, 1853, that some fifteen years before, he had, for osteo-sarcoma, extirpated the entire bone, and that his patient died a week afterwards, during his absence from town, in a supposed epileptic fit. Dr. Signoroni, of Padua, is reported (*Phil. Med. Exam.*, vol. vii, 1844, p. 96,) to have exhibited to the Medical Congress of that city, September 27, 1842, a patient, from whom, by successive operations, he had removed the entire lower jaw affected with osteo-sarcoma. The patient was then in perfect health. Mr. William Hetling, surgeon to the Bristol Infirmary, England, reported in the *Transactions of the Provincial Medical and Surgical Association*, 1833, p. 277, a case of very extensive osteo-sarcoma of the lower jaw, in which the greater part of that bone was removed, and in this report he makes the following statement: "Mr. Liston, of this city, lately removed the whole lower jaw in a case of this kind; and recovery would certainly have taken place had not an attack of the erysipelatous inflammation, then epidemic, supervened, and proved fatal."

For many years, Walther, of Bonn, has had the credit of having successfully removed the entire lower jaw, and as his claims have been questioned by some surgeons, we insert the following extract from a letter addressed by his nephew, Dr. J. E. Weber, to Dr. Perkins, of New York:

"Suffice it to say, that I myself am acquainted with eye-witnesses, yet living, who saw the case before the operation, during the operation, and after the operation and subsequent recovery, and there is at this moment in the hands of the elder son of Walther, a distinguished physician at the capital of Bavaria, a written account, minute in its details, affording a complete history of the case; which report, written by himself, at the request of his father a few days subsequent to the removal of the bone, will be published among the collected papers of Dr. Walther, which his family are about giving to the world."

I have been informed by Dr. Mott, that he has examined an individual who stated that his entire lower jaw had been removed by Mr. Hutton, of Dublin.

The most extraordinary operation on record is unquestionably that reported by Professor Syme, in the *Edinburgh Medical and Surgical Journal*, vol. xxx, 1828, p. 286. The illustrations there given present truly a frightful picture:

"The mouth was placed diagonally across the face, and had suffered such monstrous distortion as to measure fifteen inches in circumference. The throat of the patient was almost obliterated, there being only about two inches of it above the sternum, so that the cricoid cartilage of the larynx was on a level with that bone. When the tumor was viewed in profile it extended eight inches from the front of the neck. It completely filled the mouth, and occupied all the space below it from jaw to jaw. The tongue was thrust out of its place, and lay between the teeth and cheek of the right side," &c.

The jaw was removed from the right articulation to the left angle, and had a speedy recovery. The tumor weighed 4½ lbs.! In the 38th volume of the same journal, (1832,) Professor Syme states (p. 321) that the patient continued quite well, "masticating and articulating perfectly, and having nothing very disagreeable in his appearance." On the same page he refers to a preparation removed after death by Dr. Martin, of Chatham, in which the mass protruding from the patient's mouth measured at its neck twenty-one inches in circumference, and weighed 8 pounds!

I might notice in detail the successful operations of Cusack, O'Shaughnessy, Ackley, and others, in which nearly the whole bone was removed for osteo-sarcoma; but these are already familiar to the surgeon.

We will only add, that if in the terrible operation performed by Professor Syme, as well as by myself, but a few ounces of blood were lost, surely in operations of less magnitude in this region, the ligature of the primitive carotid must be unnecessary.

I have referred to the cases in which the entire lower jaw has been removed for necrosis, and, in connection with this subject, I would remark that I have in my possession the entire jaw affected with that form of the disease produced by the action of phosphorus among those engaged in the manufacture of lucifer matches. The operation was performed by Dr. Marsh, of Cincinnati, some three years since, at the infirmary; but the patient survived it only a few months. This case was, therefore, prior to that in the practice of Dr. James R. Wood, which was reported in the May number of the *New York Journal of Medicine.* Dr. Marsh's patient came from Nuremberg, Germany, a place of considerable notoriety in consequence of the prevalence of the phosphorus disease.—*Am. Jour. Med. Sc.*

## Figures and Tables

**Figure f1:**